# Pyrosequencing Analysis Reveals High Population Dynamics of the Soil Microcosm Degrading Octachlorodibenzofuran

**DOI:** 10.1264/jsme2.ME14001

**Published:** 2014-12-10

**Authors:** Wei-Yu Chen, Jer-Horng Wu, Juu-En Chang

**Affiliations:** 1 Department of Environmental Engineering, National Cheng Kung University, No. 1, University Road, East District, Tainan City 701, Taiwan (R.O.C.)

**Keywords:** Population Dynamics, Bioremediation, Octachlorodibenzofuran, Pyrosequencing

## Abstract

A deeper understanding of the microbial community structure is very important in bioremediation for polychlorinated dibenzo-*p*-dioxins/dibenzofurans (PCDD/Fs). However, this has been insufficiently addressed in previous studies. To obtain more information, we pyrosequenced the V4/V5 regions of the 16S rRNA genes of bacterial communities transited from polluted soil to batch microcosms that rapidly degraded high concentrations of octachlorodibenzofuran (OCDF). The analysis results contained an average of 11,842 reads per sample, providing the first detailed description of bacterial communities associated with PCDD/Fs. The community composition markedly changed to be concomitant with the degradation of OCDF, indicating that a distinctive population structure developed rapidly in the microcosm. Although oxygen gas was provided weekly to the microcosm, the growth of potential degraders, *Sphingomonas*, *Pseudomonas*, *Rhodococcus*, and *Clostridium*, was observed, but in consistently low quantities. While anaerobic *Sedimentibacter* initially emerged as an abundant pioneer, several aerobic participants, such as the genera *Brevundimonas*, *Pseudoxanthomonas*, and *Lysobacter*, exhibited a large increase in their 16S rRNA gene copies within the timeframe, which showed a temporal population dynamic, and indicated their collaborative contributions to the degradation of OCDF under hypoxic conditions. These results have provided a deeper insight into the microbial community structure and population dynamics of the OCDF-degrading microcosm.

Polychlorinated dibenzo-*p*-dioxins/dibenzofurans (PCDD/Fs), the impurities formed during the manufacture of chloropesticides or incineration of industrial and domestic waste, are hazardous pollutants of great concern (31). Near-fully and fully chlorinated congeners are highly resistant to microbial degradation and are typically the most predominant among PCDD/Fs in the environment (39, 42).

Various bacteria have been reported to catabolize or transform PCDD/Fs, *e.g.*, *Pseudomonas*, *Rhodococcus*, and *Sphingomonas* spp. as aerobic degraders and *Dehalococcoides* spp. as anaerobic dechlorinators (6, 19, 25, 45). The microbial degradation of PCDD/Fs can be initiated by the dioxygenation of an aromatic ring or reductive dechlorination depending on the redox level of the surrounding environment and microbial populations involved (7, 14). Under aerobic conditions, dioxygenation products can then be mineralized in the TCA cycle (20). A previous study reported that dibenzofuran may potentially be degraded under anaerobic conditions (36). However, information regarding the anaerobic catabolism of dechlorinated congeners is limited (24, 29). Although the exact degradation mechanism for hepta- and octa-chlorinated DD/Fs currently remains unclear, it is thought to require dechlorination prior to oxidative decomposition.

Soil contaminated with PCDD/Fs potentially contains microbial and genetic resources associated with the degradation of PCDD/Fs. We recently developed a microcosm that rapidly degraded octachlorinated DD/F, especially octachlorodibenzofuran (OCDF), by incubating soil taken from a chronological site contaminated with high concentrations of PCDD/Fs (10). A cloning library analysis was used to investigate the microbial community structure of the microcosm; however, the resulting data failed to detect several OCDF-degrading strains obtained. This disagreement may have been due to a low detection capability to reveal functional microorganisms at low abundance. Based on ours and other studies (10, 23, 24, 26, 46), the microbial community in the PCDD/Fs-degrading microcosm has yet to be sufficiently addressed. Such yet-to-be-improved knowledge is crucial for elucidating the microbial ecology related to the degradation of PCDD/Fs in soil environments and bioremediation of PCDD/Fs-contaminated sites.

Cost-effective and powerful high-throughput sequencing techniques have been developed to identify large-scale microbial phylotypes and have the potential to detect rare microbial populations in samples (22, 38). In the present study, we applied 454 pyrosequencing to examine microbial diversity in soil samples from a PCDD/Fs-contaminated site and resolve changes in the microbial community structure associated with the degradation of high concentrations of OCDF in the microcosms. To the best of our knowledge, this is the first study to examine microbial diversity in PCDD/Fs-contaminated soil and the population dynamics associated with the degradation of PCDD/Fs using deep sequencing.

## Materials and methods

### Soil samples

Soil samples (10–30 cm depth) were collected from a PCDD/Fs-contaminated site in the An-Shun area (120°07.467′E, 23°02.173′N) near the seacoast in Northwest Tainan city, Taiwan. Based on a large-scale investigation of the distribution of PCDD/Fs in the area, samples containing PCDD/Fs at low and high concentrations were selected and used in this study, and were named AS-O and AS-G, respectively. Soil in this area was slightly alkaline (pH, 7.5–8.5), salty (chloride, 1.42 g kg^−1^), and had a sandy loam texture with moisture, total organic carbon, nitrogen, and available phosphorous of approximately 9.8%, 1.2%, 0.1%, and 2.23 mg kg^−1^, respectively. Highly chlorinated dibenzo-*p*-dioxins and dibenzofurans (namely octa- and hepta-chlorinated congeners) contributed to >90% of the total toxicity equivalency in an averaged level of 158.2 μg TEQ kg^−1^ soil dry weight.

### Construction of AS-G and OCDF microcosms

**Fig. 1:**
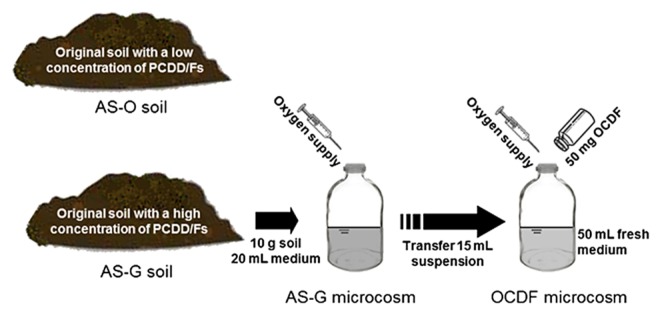
A schematic diagram showing the soils and microcosms examined in the present study.

We attempted to construct an aerobic PCDD/Fs-degrading microcosm by aerobically incubating AS-G soil with mineral medium, and designated it as the AS-G microcosm (Fig. 1). The AS-G microcosm was prepared by loading 10 g (wet weight) of AS-G soil (20-mesh sieved) and 20 mL of medium containing minerals and the yeast extract (Table S1) in 125-mL serum bottles. The headspaces were flushed with a pure oxygen stream for 3 min, capped with Teflon-coated rubber septa, and subsequently sealed with aluminum caps to maintain a closed condition at 1.5 atm of oxygen pressure. The bottles were prepared in duplicate and incubated at 28°C for 4.5 months in the dark in an orbital shaker that provided excellent mixing action (300 rpm).

To further enrich the PCDD/Fs degraders, the AS-G microcosm was partially transferred to fresh medium containing OCDF, and this microcosm was specified as the OCDF microcosm. The OCDF microcosm was prepared by transferring 15 mL of the suspension of the AS-G microcosm to bottles with 50 mL of fresh medium and 50 mg of OCDF powder. These bottles were opened weekly for sampling and oxygen replenishment. In each sampling, 2 mL of suspensions were withdrawn using glass pipettes while oxygen was supplied, as described above. The samples for microbial analysis were stored at −20°C, while those for the PCDD/Fs analysis were air-dried and stored at 4°C.

### PCDD/Fs analysis

OCDF and congeners in the samples were Soxhlet extracted with toluene, purified, and analyzed using the isotope dilution high-resolution GC-MS method, as described previously (27).

### DNA recovery and quantitative PCR analysis of the 16S rRNA gene

Total DNA was recovered from soil and microcosm samples with the Mo Bio PowerSoil DNA isolation kit (Carlsbad, CA) and purified using the DNA Clean-Up System (Promega, USA). The concentrations of double-stranded DNA were determined using a TBS-380 Mini-Fluorometer (Turner BioSystem, CA, USA) with a PicoGreen dsDNA quantitation reagent (Molecular Probes, Oregon, USA). A SYBR Green quantitative PCR (qPCR) analysis of the bacterial 16S rRNA gene was performed on an FX96 real-time PCR system (BioRad, US). The qPCR reaction mixture (total volume, 20 μL) contained 1X SYBR^®^ Premix Ex Taq™ (Takara, Japan), 0.2 mM of each *Bacteria*-specific primer (1087F, 5′-GGTTAA GTCCCSYYACGAGC-3′; 1392R, 5′-ACGGGCGGTGTGTAC-3′), and 2 μL of genomic DNA. The PCR temperature program was set at 95°C for 3 min, followed by 45 cycles at 95°C for 5 s, 56°C for 15 s, and 72°C for 15 s. qPCR was performed in triplicate for each sample. The gene copy numbers of the samples were calculated using a linear comparison of threshold cycles obtained in each qPCR run with a calibration curve that was obtained using 10-fold serial dilutions of known plasmid DNA concentrations, ranging from 10^2^ to 10^7^ gene copies per nanogram of template DNA. Nuclease-free water or a plasmid without an insert was employed as the negative control. The accuracy of the qPCR assay was confirmed using both melting curve analysis and agarose gel electrophoresis.

### Barcoded PCR and 454 pyrosequencing of the 16S rRNA gene

Prior to pyrosequencing, the bacterial 16S rRNA genes were amplified with universal forward 562F (5′-Fusion A-Barcode-AYTGGGYDTAAAGNG-3′) and reverse 906R (5′-Fusion B-Barcode CCGTCAATTYYTTTRAGTTT-3′) primers, which targeted for amplification of the variable regions V4/V5 of the 16S rRNA gene (12). The PCR reaction mixtures comprised 10 ng of genomic DNA, 200 nM (each) of the forward and reverse primers, 0.2 mM of dNTP (each), 1X FastStart Buffer (Roche, Indiana, USA), and 1.25 U of FastStart HiFi Polymerase (Roche, Indiana, USA) in a volume of 24 μL. Reactions were run in a 9700 thermal cycler (Applied Biosystems, Foster, CA, USA) by denaturing DNA for 3 min at 94°C, followed by 40 cycles of 15 s at 94°C, 45 s at 50°C, 1 min at 72°C, and final extension at 72°C for 8 min. The presence of amplicons was confirmed using gel electrophoresis on a 1.5% agarose gel. The PCR amplicons were purified using Agencourt AMPure XP Reagent (Beckman Coulter Inc., Beverly, Massachusetts, USA) and subsequently quantified using an Agilent Bioanalyzer (Agilent Technologies Inc., Deutschland, Waldbronn, Germany). Equimolar amounts of the PCR amplicons were mixed in a single tube, and pyrosequencing was performed using a 454 GS Junior (Roche Applied Science, Branford, CT, USA).

### Pyrosequencing data analysis

The sequence reads were sorted based on their respective barcodes into 8 libraries. By applying 454 pyrosequencing, we obtained 94,732 qualified sequences with a distribution of 10,109– 14,775 sequences on each sample (Table 1). To minimize the effects of random sequencing errors, the raw sequence reads were checked for their quality. Low-quality sequences (*i.e.*, length <150 bp) were removed from the libraries. After deleting the primer, barcode, and adaptor sequences, the qualified 16S reads had a mean read length of 315–350 bp (Table S2) and a quality score (Q value) of 37. The reverse sequences were complemented on the RDP Pipeline Initial Process. The qualified sequences were then aligned based on the RDP Infernal and phylogenetically assigned using the RDP Classifier at a 95% confidence level (44). The cluster files were subsequently employed to generate rarefaction curves, which showed an accumulating trend on operational taxonomic units (OTUs) defined at the 97% similarity level with respect to the total number of sequence reads (Fig. S1). To account for the variation of a bacterial group, sequence abundances were calculated by multiplying the relative OTU frequency with total sequence reads in each of the samples.

### Nucleotide sequence accession numbers

The nucleotide sequences obtained in this study were deposited at the EMBL-EBI European Nucleotide Archive under the study accession number PRJEB4603 (http://www.ebi.ac.uk/ena/data/view/PRJEB4603).

## Results

### Microbial communities of uncultivated soil with low and high concentrations of PCDD/Fs

The soil in the An-Shun site had been contaminated with high concentrations of PCDD/Fs for decades (27). To examine the effects of PCDD/Fs on the bacterial community structure, soil samples containing PCDD/Fs at high and low concentrations, *i.e.*, AS-G and AS-O, respectively, were obtained from close sampling locations. As shown in Table 1, the AS-O soil had PCDD/Fs (toxicity equivalency, 165.0 ng TEQ kg^−1^; OCDF concentration, 43.1 nmol kg^−1^) below the regulation standard (1.0 μg TEQ kg^−1^). The AS-G soil contained high concentrations of PCDD/Fs (1583.6 μg TEQ kg^−1^), 24.1% (834.5 μmol kg^−1^) of which was contributed by OCDF. This concentration was markedly higher (19,407-fold) than that of the AS-O soil. Table 1 shows that the copies of the bacterial 16S rRNA gene in the AS-G and AS-O soils were observed at magnitudes of 10^6^ and 10^7^, respectively, which revealed the influence of the PCDD/Fs contamination on the density of the bacterial population.

The RDP Classifier was used to assign the 16S reads into different OTUs (cut-off, 97% sequence similarity), yielding 3,227 and 1,783 OTUs for the AS-O and AS-G soils, respectively. The rarefaction curves did not appear to reach a plateau, even though 10,353–10,720 reads had been sequenced (Fig. S1), which indicated that the microbial community in soil was extremely complex. To assess the internal (within-sample) complexity of the individual bacterial community, various indices of microbial diversity were calculated. The values of the Shannon-Weaver index (*H*), Chao-1, and Evenness indices were higher for the AS-O soil than for the AS-G soil. Combined with the reduced bacterial population in the AS-G soil, the less diversity of the AS-G soil was attributed to a reduction in bacteria intolerant to high concentrations of PCDD/Fs.

**Fig. 2:**
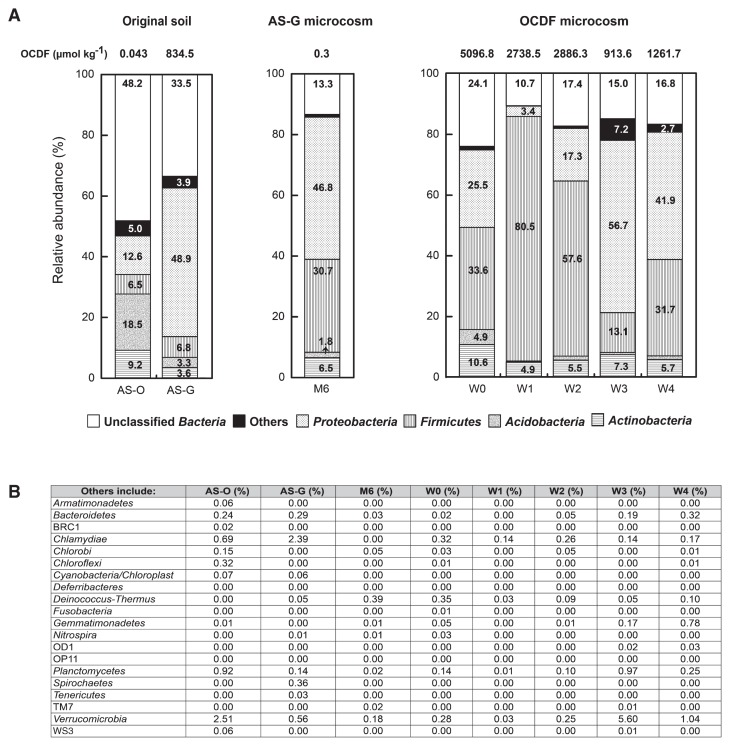
Bacterial compositions evaluated at the phylum level. (A) Bacterial communities were dominated by *Proteobacteria*-, *Firmicutes*-, *Acidobacteria*-, and *Actinobacteria*-related populations. The numbers indicated the sequence abundance in percentages. Phyla detected with low sequence abundance were grouped as “others”, and their exact percentages are listed in (B).

The RDP Classifier was also employed to assign the detected sequences to various levels of phylogenetic bacterial taxa. The results obtained showed that at the phylum level, 15 and 13 bacterial phyla in RDP were detected for the bacterial communities of the AS-O and AS-G soils, respectively. Among these, as shown in Fig. 2A, the members of *Acidobacteria*, *Proteobacteria*, *Actinobacteria*, and *Firmicutes*, accounting for a relative abundance of 18.5%, 12.6%, 9.2%, and 6.5% of all sequences, respectively, were predominant in the bacterial community of the AS-O soil. The abundant members in the AS-G soil were also affiliated with the 4 phyla. However, *Proteobacterial* populations (36.3%) were markedly larger and *Actinobacterial* (5.6%) and *Acidobacterial* (15.2%) populations were slightly smaller in the AS-G soil than in the AS-O soil. As shown in Fig. 2B, the members belonging to the remaining 11 and 9 bacterial phyla were detected at a relatively low frequency (<2.51% in corresponding libraries). The *Bacteroidetes*, *Chlamydiae*, *Cyanobacteria*, *Planctomycetes*, and *Verrucomicrobia* could be detected in both bacterial communities. The populations of *Armatimonadetes*, *Chlorobi*, *Chloroflexi*, and *Gemmatimonadetes*, as well as not-yet-cultured populations in candidate divisions BRC1 and WS3 could only be detected in the AS-O soil, whereas the populations belonging to *Deinococcus-Thermus*, *Nitrospira*, *Spirochaetes*, and *Tenericutes* were only detected in the AS-G soil.

### PCDD/Fs degradation and microbial community in the AS-G microcosm

During a 6-wk incubation in the AS-G microcosm, the degradation of approximately 834.1 μmol kg^−1^ of OCDF accompanied the rapid depletion of oxygen (18% oxygen remained). The lower-chlorinated congeners, such as 2,3,4,6,7,8-H*_x_*CDF increased largely from 5.6 nmol kg^−1^ to 4.7 μmol kg^−1^, suggesting the dechlorination of OCDF. However, this increment in congener concentration was not stoichiometrically equivalent to the decrease in OCDF. In the microcosm on the 6th wk, denoted as M6, the copy numbers of the bacterial 16S rRNA gene were maintained at a magnitude of approximately 10^6^ copies g^−1^ soil [dry weight], which was similar to that in the AS-G soil of the inoculum. However, microbial diversity became more complicated than that in the AS-G soil because the pyrosequencing analysis revealed an increase in the values of species richness, Shannon-Weaver index (*H*), and Chao-1 (Table 1). As shown in Fig. 2A, the frequencies of phylotypes related to *Firmicutes* and *Actinobacteria* in M6 increased to 30.7% and 6.5%, respectively, whereas those in *Proteobacteria* (46.8%) and *Acidobacteria* (1.8%) remained relatively constant.

### OCDF degradation and microbial community in the OCDF microcosm

**Fig. 3:**
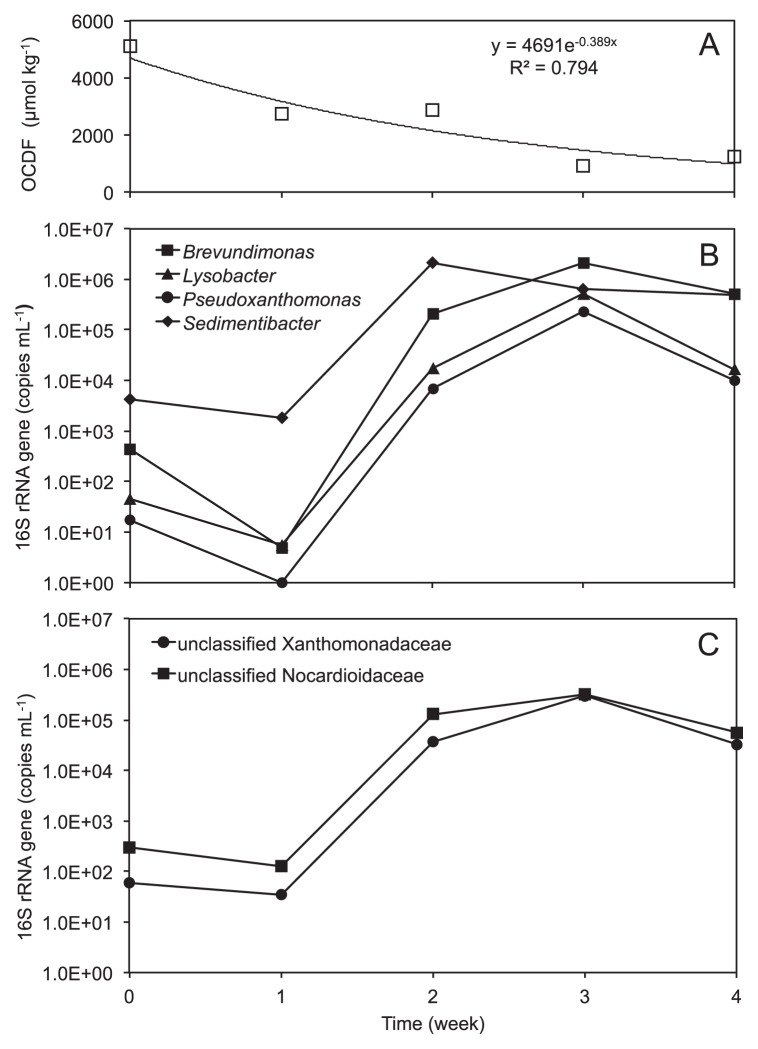
(A) Degradation of OCDF in the transferred microcosm. An exponential approximation curve was used to indicate the degradation trend. Concentration variations in the 16S rRNA gene related to the bacterial genera (B) and unclassified bacterial groups (C).

Fig. 3A shows OCDF degradation activity in the OCDF microcosm, which was constructed by cultivating a suspension of the AS-G microcosm with OCDF for 4 wk. As shown in Fig. 3A, under the conditions of the transferred microcosm, OCDF could be degraded at a high rate of approximately 949.5 μmol kg^−1^ wk^−1^ without the accumulation of lower chlorinated congeners.

Since only the supernatant was used as an inoculum, the q-PCR analysis showed that the OCDF microcosm was established with a diluted bacterial concentration of approximately 1.75±0.38×10^4^ copies mL^−1^ in the sample W0 on the day of transferal (Table 1). Regarding the weekly samples designated according to the week number, *i.e.*, W1–W4, bacterial 16S rRNA genes declined to 3.10±0.20×10^3^ copies mL^−1^ in the W1 sample. This decrease in the bacterial concentration may, in part, be attributed to the toxicity of the high OCDF dose. However, rapid bacterial growth subsequently proceeded because the 16S rRNA gene quantity greatly increased to 4.84±0.94×10^6^ copies mL^−1^ in the W2 sample, and plateaued at a magnitude of 10^6^ in the W3 and W4 samples. The results of the qPCR analysis indicated that bacterial populations multiplied in the OCDF microcosm.

As shown in Fig. 2A, the pyrosequencing analysis revealed that the microbial community markedly changed in the OCDF microcosm during the incubation. In the beginning of the OCDF microcosm, the phylotypes of particular phyla, *Actinobacteria* (10.6%), *Acidobacteria* (4.9%), *Firmicutes* (33.6%), and *Proteobacteria* (25.5%), were abundant. Among the 4 samples (*i.e.*, W1–W4), the 16S rRNA gene sequences of *Firmicutes*-related populations increased to the maximum percentage of 80.5% for the W1 sample, while *Proteobacteria*-related populations were more dominant in the W3 sample, showing a rapid alternation in the two populations.

### Comparison of bacterial community structures

Fig. S2 shows a heat map that was obtained based on the sequence frequencies of the top 10 abundant taxa that covered 95.6–99.5% of all sequences. The hierarchical heat map analysis clearly identified 3 groups, indicating similar degrees of bacterial community structures among the samples. Group I was clustered by samples W0, M6, W4, and W3. The M6 and W0 samples shared a highly similar community structure that resembled that of the W4 and W3 samples, demonstrating a repeatable succession of bacterial compositions. However, differences were observed among the 4 bacterial communities. The W0 and M6 sample communities were primarily distinguished by the greater prevalence of *Clostridiales*-related populations, whereas the W3 sample community could be characterized by the dominance of *Xanthomonadales*- and *Caulobacterales*-related populations. Group II comprised non-cultivated soil samples (AS-O and AS-G), in which the dominant populations were clearly dissimilar to all other populations in the microcosm samples. Group III comprised the W1 and W2 samples and distantly branched from Groups I and II. Based on the cluster heat map analysis, the populations related to the *Clostridiales* order were the main contributors to the differences observed in bacterial community structures.

To investigate the relationship between bacterial community structures at a refined level, we conducted a principal component analysis (PCA) with the detection frequencies of the 198 genera data obtained from 8 samples (Fig. S3). The PCA results were generally consistent with those of the clustered heat map analysis, but grouped the bacterial communities in a slightly different manner. This difference may have been caused by the different resolution and extraction of data in the clustering analysis. The vector projection in the PCA biplots revealed that bacterial communities in the M6 and W0–W4 samples strongly correlated with the genera *Sedimentibacter* and *Brevundimonas*. These results also suggested that the *Lysobacter*- and *Pseudoxanthomonas-*related populations appeared to have a role in these samples (Fig. S3). Since the qPCR analysis indicated prominent bacterial growth in the OCDF microcosm (Table 1), we further estimated the 16S rRNA gene copies of these four populations (total copies of the bacterial 16S rRNA gene × relative abundance of a corresponding group in the library). As shown in Fig. 3B, the results obtained revealed a large increment in the corresponding 16S rRNA gene copies up to 10^2^–10^6^-fold. Following the *Sedimentibacter*, the populations related to *Brevundimonas* spp., accounting for 27.5% of all sequences, became the most predominant in the community. The correlation analysis suggested that the degradation of OCDF strongly correlated with the population abundance of *Brevundimonas*, *Lysobacter*, and *Pseudoxanthomonas* at correlation coefficients of 0.816, 0.694, and 0.704, respectively. In addition to the four populations, two unclassified groups were detected in the *Xanthomonadaceae* and *Nocardioidaceae* families with notable sequence percentages (1.67%–4.19%) and similar temporal dynamics (Fig. 3C). However, due to the short read lengths of sequences, their exact identities could not be ascertained.

### *In silico* screening of yet-isolated PCDD/Fs degraders

*In silico* screening of possible degraders was conducted in all the samples used in this study by screening for bacterial genera reported to degrade PCDD/Fs and relative compounds. Table 2 shows the populations of 10 genera as possible degraders detected in the AS-O soil, with each accounting for <1.47% of all sequences, or with 16S rRNA gene quantities in the range of 10^3^–10^4^ copies g^−1^ soil [dry weight]. Among the 6 aerobic populations, *Sphingomonas* relatives were constantly detected in the OCDF microcosm in the range of 10^1^–10^4^ copies mL^−1^. Negligible amounts of the remaining 5 aerobes were observed. In the OCDF microcosm, *Pseudomonas* and *Rhodococcus* spp. could proliferate to 10^3^ copies mL^−1^, corresponding to a sequence abundance of 0.06% in the W3 sample. The 2 populations detected were affiliated with the facultative dehalorespirers, *Anaeromyxobacter* and *Geobacter*. However, they were only observed in the non-cultivated soil samples. We detected 2 anaerobic populations related to the groups of *Clostridium* XI and *Clostridium* sensu stricto that were previously shown to perform reductive dehalogenation co-metabolically (9, 16, 21). Their prevalence was similar to that of *Sphingomonas*. Due to their degradative potentials and medium-to-high correlation to the degradation of OCDF (correlation coefficient =0.569–0.806), these 3 populations likely played a critical role in the OCDF microcosm. In the present study, no sequence among any library was associated with any known obligate dehalorespirer.

## Discussion

In the present study, we used a high-throughput pyrosequencing technique to contrast the microbial community structures in soil with low and high concentrations of PCDD/Fs, and reported the first succession of bacterial community structures to the degradation of OCDF in batch microcosms. The results of 16S rRNA-based pyrosequencing showed that the degree of complexity associated with microbial diversity was sensitive to PCDD/Fs, and could be markedly reduced in heavily contaminated soil, indicating the impact of PCDD/F toxicants on the soil microbiome. We examined populations related to 4 specific phyla (*Proteobacteria*, *Actinobacteria*, *Firmicutes*, and *Acidobacteria*) that have been typically observed in healthy soil (15, 34), and elucidated differences in the dynamic tendencies of how they responded to the enrichment processes with OCDF.

Although the studied congener OCDF, a fully chlorinated furan, is thought to be highly resistant to microbial degradation, we observed the degradation of OCDF in soil and freshly added to the microcosms, with faster degradation rates (Fig. 3A) than those of other PCDD/Fs reported previously (15, 18, 33, 46). This result was attributed to the levels of hundreds of parts per million (ppm) used in this study being markedly higher than those of parts per billion (ppb) to several ppm used in previous studies. The pyrosequencing and qPCR analyses performed in the present study revealed that the 16S rRNA gene showed the development progress of a distinct bacterial community, which could be shaped promptly during degradation.

The bacterial populations with abundant sequence reads displayed different development dynamics in the OCDF microcosm. The sequence percentages of the *Actinomycetales-*related populations that were predominant in the phylum *Actinobacteria* remained relatively constant. The *Caulobacterales*-, *Xanthomonadales*-, and *Clostridiales-*related populations were subject to marked variations (Fig. S2). A detailed observation of weekly developments at the genus level showed that the *Sedimentibacter*-related populations of order *Clostridiales* could strongly proliferate during the first 2 wk of the degradation of OCDF, while the populations related to *Xanthomonadales* (such as *Lysobacter* and *Pseudoxanthomonas*) and *Caulobacterales* (such as *Brevundimonas*), as well as *Pseudomonadales* (such as *Pseudomonas*) and *Sphingomonadales* (such as *Sphingomonas*) in turn succeeded in the late stage (Fig. 3B and Table 2). In addition to a distinctive community structure, the marked change in the bacterial populations suggested a staged dynamic process, and may have correlated with the degradation of OCDF in the microcosm.

In the microcosm experiments to prepare redox environments with pure oxygen, it was of novel value that, in addition to the aerobes, we observed the growth of several anaerobic fermenting populations such as *Sedimentibacter* and *Clostridium*. Several studies have indicated that PCDD/Fs could be favorable to reductive dechlorination processes under conditions that are not strictly anaerobic (24, 46). However, other studies reported that aerobic degraders with aromatic ring-cleaving dioxygenases were active under oxygen-limiting conditions because the relevant mRNA transcript could be strongly expressed in the hydrocarbon-contaminated aquifer (41). Although this may have been due to oxygen gradients, oxygen-limiting or hypoxic conditions should account for the actual redox level of a solution environment, hence facilitating the co-existence of (facultative) aerobic and anaerobic populations to achieve the combined dechlorination and oxidative degradation of OCDF. This is different from the situations under obligate anaerobic and aerobic conditions, in which reductive dechlorination and oxidative degradation take place, respectively (14). Such an understanding can be applied to the development of a bioremediation process for removing PCDD/Fs pollutants from soil. For example, air and nutrient supplements can be provided in an intermittent manner in order to maintain hypoxic conditions in the batch reactor, allowing for the establishment of a capable microbial community to achieve the degradation of PCDD/Fs (33).

The degradation of OCDF was previously suggested to be initiated by an initial dechlorination process to free several chlorine-substituted positions downstream for the hydroxylation and cleavage of aromatic rings (24); therefore, we specifically examined pyrosequencing data in order to identify the possible participants. Obligate anaerobes (*e.g.*, *Dehalococcoides* and *Dehalobacter*) that have previously been shown to reductively transform PCDD/Fs and chlorinated aromatic compounds (13, 29) were not detected in this study, although the depth of pyrosequencing reached 10^4^ per sample (total > 6×10^4^). A PCR analysis with the primers DHC793F-1492R (46) and 8F-Dre645R (37), which are specific to the 16S rRNA of *Dehalococcoides* populations, also indicated negative detections with all DNA templates in this study. These results suggested that the reductive dehalorespiration mechanism did not significantly contribute to the degradation of OCDF in the microcosm, which differs from previous findings in which *Dehalococcoides*-like dechlorinators at a detectable abundance have been suggested to play a critical role in the degradation of PCDD/Fs in anaerobic microcosms (0.1% of all bacteria) (46) and semi-aerobic composts (0.005%–0.016% of all bacteria) (33).

Nevertheless, the evaluation of pyrosequencing data at the genus level identified the growth of several anaerobic bacteria, such as *Clostridium* XI and *Clostridium* sensu stricto (Table 2), which were previously reported to be capable of co-metabolic reductive dehalogenation (1, 28). In addition, certain strains under the genera *Pseudomonas*, *Rhodococcus*, and *Mesorhizobium* that were found to dechlorinate tetrachlorobenzene, chlorinated biphenyl, and chlorinated dioxins (2, 3, 10, 35) were also detected in this study.

The anaerobic *Sedimentibacter* spp., which favors anaerobic habitats with rich organic carbons, a high salt content, and metals (8, 40), were detected (4.1%) in the original soil at the site, and were in high sequence abundance during the operation of microcosms (~58.6%). The *Sedimentibacter* spp. have been detected in dechlorinating co-cultures (4, 17, 47). However, it was recently predicted that the *Sedimentibacter* genome contains no reductive dehalogenase homologue-encoding genes (30), and, furthermore, no dechlorination activity was observed for β-hexachlorocyclohexane or the chloroethenes (11, 43). A metagenomic analysis indicated that *Sedimentibacter* provided adenosyl-cobalamin and various amino acids required by the dechlorinator partners in co-cultures (30). Therefore, *Sedimentibacter* is not expected to emerge as a pioneer population with relatively high sequence abundance in the community when a large amount of OCDF is degraded under oxygen-containing conditions. Moreover, *Sedimentibacter* spp. are fermentative bacteria and require yeast extracts for growth (5). We assumed that the yeast extract (and soil organics) that was added to the medium subsequently stimulated the growth of *Sedimentibacter*, which may, in turn, have provided nutrients and a carbon source to stimulate the activities of other community members.

The pyrosequencing analysis detected several relevant genera known as PCDD/Fs degraders such as *Sphingomonas*, *Pseudomonas*, *Rhodococcus*, *Bacillus*, and *Mesorhizobium*, and their proportions, to a lesser extent, were varied in spite of their low sequence abundances (0.02%–1.47%). Unlike those with known degradation potentials, several genera such as *Lysobacter*, *Pseudoxanthomonas*, and *Brevundimonas*, which were seldom observed in relation to the degradation of PCDD/Fs, could exhibit marked and temporary increases in sequence abundance, which strongly correlated with the degradation of OCDF (Fig. 3). Their dominance could be supported in accordance with the results obtained using a clone library analysis combined with a hierarchical oligonucleotide primer extension method (10). We previously obtained a pure culture of *Brevundimonas basaltis* (strain M23) that contained ring-hydroxylating dioxygenases genes and was able to grow in OCDF-containing basal medium (10). The ecological importance of these microorganisms in the microcosm could be predicted based on the genetic and physiological traits associated with the degradation of PCDD/Fs. However, we only analyzed 16S rRNA gene amplicons retrieved from genomic DNA; therefore, their exact functions and interactions with other populations in the complex microcosm remain relatively unknown and should be examined in future studies.

## Conclusion

We herein demonstrated that the high-throughput pyrosequencing approach used could characterize the microbial diversities of soil and microcosms, thereby providing an insight into changes in microbial community structures with the degradation of OCDF. Although this technique only resolved nucleotide sequences to the genus level, with increasing improvements in read lengths, it has the potential to detect bacterial populations at a refined resolution. Although the exact redox range was unknown, our results suggested that the effective degradation of OCDF required diverse anaerobic and aerobic populations that co-varied in a microcosm under hypoxic conditions, thereby providing a lead to improve bioremediation methods for PCDD/Fs-contaminated soil.

## Supplementary Information



## Figures and Tables

**Table 1:** Summary of 16S rRNA gene quantity, numbers of pyrosequences, and estimated diversity indices for samples analyzed in this study.

Sample ID	Sample Type	16S rRNA^a^	Read^b^	Richness^c^	*H*^d^	Chao 1^e^	Evenness^f^
AS-O	Original soil with low concentrations of PCDD/Fs	1.15±0.97×10^7^	10,720	3,227	7.28	5,871	0.90
AS-G	Original soil with high concentrations of PCDD/Fs	2.52±0.34×10^6^	10,353	1,783	6.07	3,419	0.81
M6	AS-G microcosm at the 6^th^ wk	1.09±0.19×10^6^	13,357	2,615	6.28	4,328	0.80
W0	OCDF microcosm at 0 d	1.75±0.38×10^4^	10,553	2,659	6.75	4,460	0.86
W1	OCDF microcosm at the 1^st^ wk	3.10±0.20×10^3^	14,775	2,349	6.21	3,604	0.80
W2	OCDF microcosm at the 2^nd^ wk	4.84±0.94×10^6^	13,029	3,030	6.76	4,864	0.84
W3	OCDF microcosm at the 3^rd^ wk	7.58±2.57×10^6^	11,836	2,411	6.34	4,249	0.81
W4	OCDF microcosm at the 4^th^ wk	2.19±0.44×10^6^	10,109	2,353	6.57	3,952	0.85

a (copies g^−1^ soil dry weight) for soil and slurry samples; (copies mL^−1^ suspension) for suspension samples.

b Number of qualified pyrosequences.

c Number of detected OTUs.

d Shannon-Weiner index; a higher number represents a higher degree of diversity.

e Chao 1 richness estimate; a higher number represents a higher degree of diversity.

f Evenness index

**Table 2:** Estimate of 16S rRNA gene copies of bacterial groups potentially containing the degradative capability for PCDD/Fs^a^.

Symbol^b^	Genera^c^	16S rRNA gene^d^							

		AS-O	AS-G	M6	W0	W1	W2	W3	W4
A	*Sphingomonas*	1.50×10^4^	3.78×10^3^	N.D.	1.28×10^2^	4.56×10^1^	4.84×10^2^	2.12×10^4^	4.16×10^3^
A	*Pseudomonas*	1.15×10^4^	1.26×10^3^	N.D.	3.50×10^0^	1.86×10^0^	N.D.	4.55×10^3^	N.D.
A	*Rhodococcus*	2.19×10^4^	N.D.	N.D.	2.28×10^1^	0.62×10^0^	N.D.	4.55×10^3^	N.D.
A	*Bacillus*	1.15×10^4^	N.D.	N.D.	N.D.	0.62×10^0^	N.D.	N.D.	N.D.
A	*Micrococcus*	6.90×10^3^	5.04×10^3^	N.D.	N.D.	N.D.	N.D.	N.D.	N.D.
A	*Mesorhizobium*	1.50×10^4^	N.D.	5.45×10^2^	N.D.	0.62×10^0^	N.D.	N.D.	1.10×10^3^
F	*Anaeromyxobacter*	3.68×10^4^	N.D.	N.D.	N.D.	N.D.	N.D.	N.D.	N.D.
F	*Geobacter*	3.45×10^3^	8.82×10^3^	N.D.	N.D.	N.D.	N.D.	N.D.	N.D.
+	*Clostridium* XI	1.15×10^4^	N.D.	2.73×10^3^	4.90×10^1^	2.95×10^1^	2.52×10^4^	7.05×10^4^	7.23×10^3^
+	*Clostridium* sensu stricto	1.50×10^4^	2.04×10^4^	3.60×10^3^	6.83×10^1^	3.10×10^1^	2.52×10^4^	1.67×10^4^	3.94×10^3^

a As suggested by (7), (20), and (32).

b A, aerobic dioxin degrader; O, obligate dehalorespirer; F, facultative dehalorespirer; +, bacteria showing co-metabolic reductive dehalogenation.

c Studied, but not detected in this study: A: *Beijerinckia*, *Burkhoderia*, *Alcaligenes*, *Klebsiella*, *Terrabacter* O: *Dehalococcoides*, *Dehalobacter* F: *Sulfurospirillum*, *Desulfomonile*, *Desulfuromonas*, *Desulfitobacterium* +: *Propionigenium*, *Shewanella*, *Desulfobacterium*, *Acetobacterium*

d (copies g^−1^ soil dry weight) for samples AS-O, AS-G, and M6; (copies mL^−1^ suspension) for samples W0–W4.
