# Supplementary material for: Pyrosequencing Analysis Reveals High Population Dynamics of the Soil Microcosm Degrading Octachlorodibenzofuran
Source: Microbes Environ. 2014 Dec 10;29(4):393–400. doi: 10.1264/jsme2.ME14001 (PMC4262363; doi:10.1264/jsme2.ME14001)

Submitted to Microbes and Environments

Pyrosequencing Analysis Reveals High Population Dynamics of Soil Microcosm Degrading Octachlorodibenzofuran

WEI-YU CHEN, JER-HORNG WU*, and JUU-EN CHANG

Department of Environmental Engineering, National Cheng Kung University, No.1, University Road, East District, Tainan City 701, Taiwan (R.O.C.)

***Corresponding author**: E-mail: enewujh@mail.ncku.edu.tw; Tel.: +886-910385159; FAX: +886-6-2752790.

Table S1 The medium used in this study.

Composition	Concentration (g L^{-1})
Na_2HPO_4	2.2
NH_4Cl	1
KH_2PO_4	0.8
$MgSO_4 \cdot 7H_2O$	0.2
$FeSO_4 \cdot 7H_2O$	0.1
$CaCl_2$	0.1
Yeast extract	0.2

Table S2 Number of 16S pyrotags obtained for each sample.

Sample	No. of raw pyrotags	No. of pyrotags after quality control	% of raw pyrotags removed	Average length (nt)
AS-O	11,653	10,720	8.0	348
AS-G	11,218	10,353	7.7	350
M6	15,007	13,357	11.0	315
W0	12,227	10,553	13.7	323
W1	15,987	14,775	7.6	326
W2	14,532	13,029	10.3	321
W3	13,426	11,836	11.8	321
W4	11,411	10,109	11.4	325

Figure Legends

Figure S1. Rarefaction curves of OTUs clustered at 97% phylotype similarity level.

Figure S2. A heat map displaying Bray-Curtis similarities based on the sequence frequencies of the assigned OTUs at the order level is shown. A frequencies comparison is visualized, in which high percentages are indicated in light gray and low percentages are indicated in dark gray. The color-coded scale bar is shown on the left of the heat map. Clustering of the microbial communities was performed based on the taxon percentages, which are indicated on each column, and the resulting dendrogram is shown on the top panel. The order/phylum taxa used are showed on the right of the heat map. The clustering heat map was generated using the Heatplus program in the gplots package for R.

Figure S3. An ordination biplot of principal component analysis (PCA) was used to indicate the relationship among the microbial community structures at the genus level. Approximately, 51.7%–62.2% of total sequences could be classified to the genus taxa, and 198 different genera were acquired. The dataset comprising the sequence frequencies of the 198 genera taxa (cases) distributed in the 8 samples (variables) was then used for calculating the correlation matrix in PCA using statistical software Statistica 8.0 (StatSoft, USA). The resulting two projection plots of variables and cases were merged into the biplot (Ramette, A. *FEMS Microbial. Ecol*. 2007, 62: 142-160). In the biplot, only the main genera and phylogroups including *Brevundimonas*, *Sedimentibacter*, *Lysobacter*, *Pseudoxanthomonas*, GP2, and GP6 (arrows), and the microbial communities of original soils (■), AS-G microcosm (▲), and OCDF microcosms (●) were represented. The percentage of variance explained by each of the two first principal components (*i.e.*, PC1 and PC2) is listed after each axis heading. The sample definition refers to the Table 1 and Fig.1 in the main text.

Supplementary Figure S1 (Chen *et al*.)

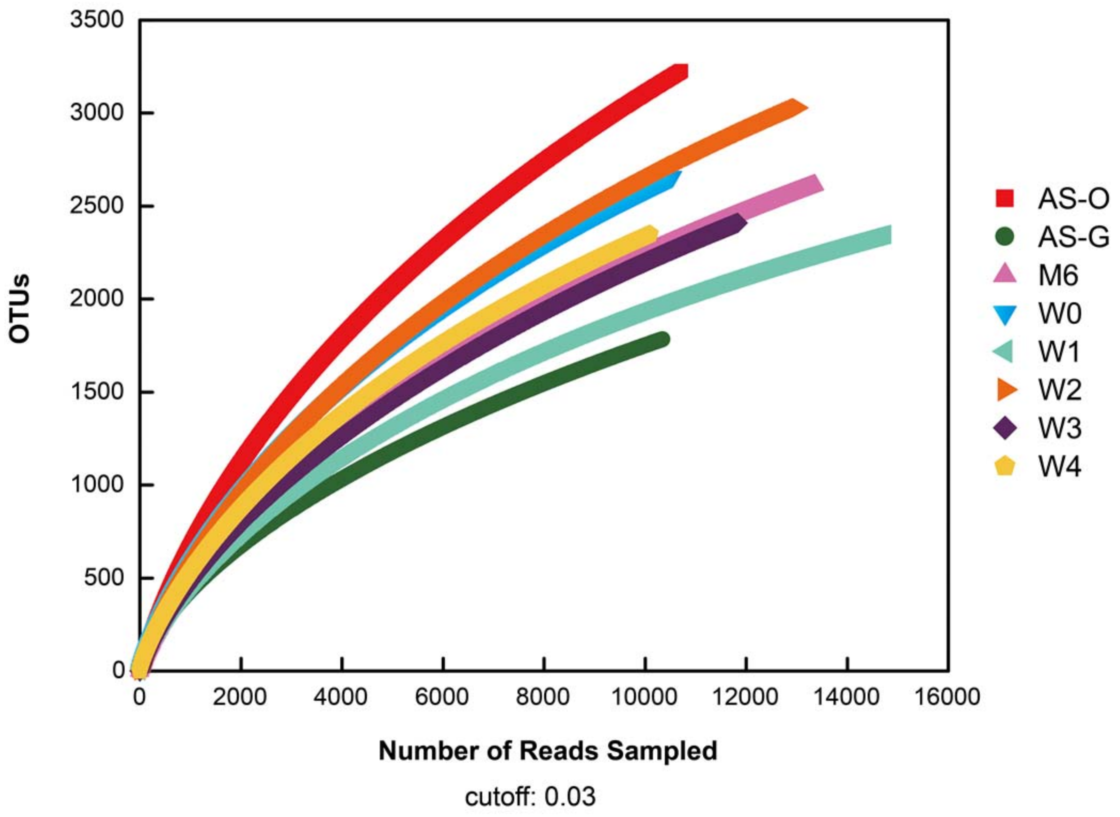

Supplementary Figure S2 (Chen *et al*.)

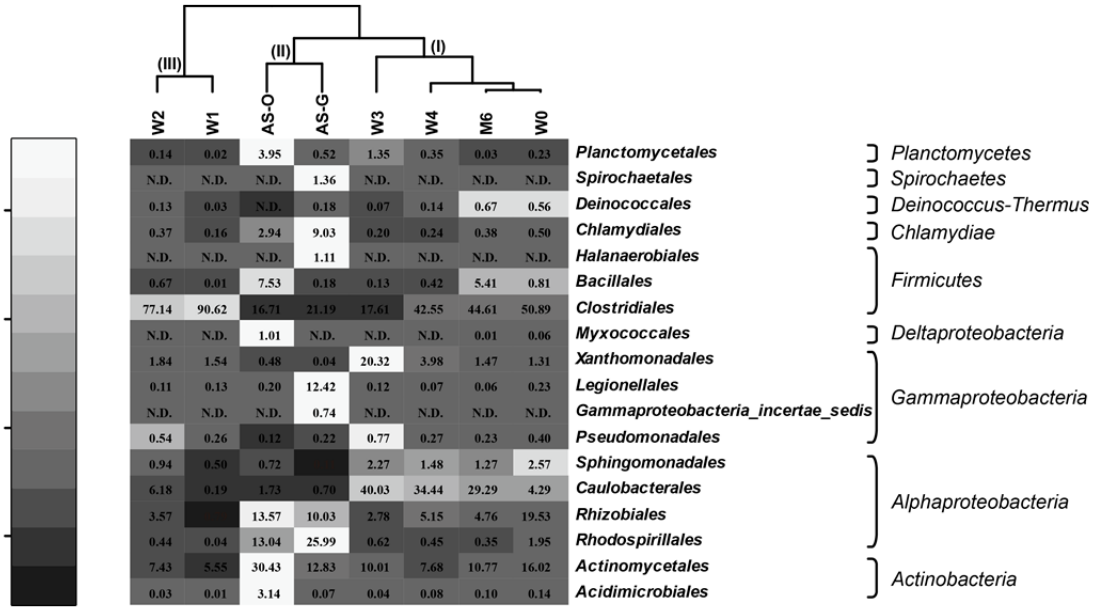

Supplementary Figure S3 (Chen *et al.*)

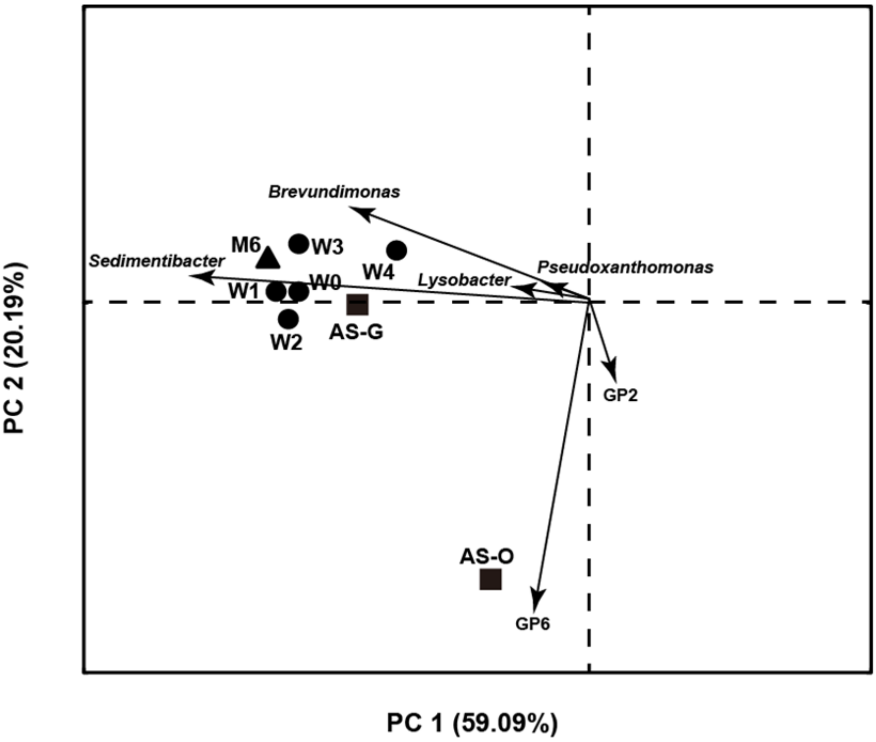

Supplement: Supplementary file 1 [file 29_393_s1.pdf]
